# Evaluating mortality in intensive care units: contribution of competing risks analyses

**DOI:** 10.1186/cc3921

**Published:** 2005-12-01

**Authors:** Matthieu Resche-Rigon, Elie Azoulay, Sylvie Chevret

**Affiliations:** 1 Medical Doctor, Biostatistics Department, Saint Louis Teaching Hospital-Assistance Publique-Hôpitaux de Paris, 1 avenue Claude Vellefaux, Paris, 75010, France; 2 Medical Doctor, Medical Intensive Care Unit, Saint Louis Teaching Hospital-Assistance Publique-Hôpitaux de Paris, 1 avenue Claude Vellefaux, Paris, 75010, France; 3 Medical Doctor, Biostatistics Department, Saint Louis Teaching Hospital-Assistance Publique-Hôpitaux de Paris, 1 avenue Claude Vellefaux, Paris, 75010, France

## Abstract

**Introduction:**

Kaplan–Meier curves and logistic models are widely used to describe and explain the variability of survival in intensive care unit (ICU) patients. The Kaplan–Meier approach considers that patients discharged alive from hospital are 'non-informatively' censored (for instance, representative of all other individuals who have survived to that time but are still in hospital); this is probably wrong. Logistic models are adapted to this so-called 'competing risks' setting but fail to take into account censoring and differences in exposure time. To address these issues, we exemplified the usefulness of standard competing risks methods; namely, cumulative incidence function (CIF) curves and the Fine and Gray model.

**Methods:**

We studied 203 mechanically ventilated cancer patients with acute respiratory failure consecutively admitted over a five-year period to a teaching hospital medical ICU. Among these patients, 97 died before hospital discharge. After estimating the CIF of hospital death, we used Fine and Gray models and logistic models to explain variability hospital mortality.

**Results:**

The CIF of hospital death was 35.5% on day 14 and was 47.8% on day 60 (97/203); there were no further deaths. Univariate models, either the Fine and Gray model or the logistic model, selected the same eight variables as carrying independent information on hospital mortality at the 5% level. Results of multivariate were close, with four variables selected by both models: autologous stem cell transplantation, absence of congestive heart failure, neurological impairment, and acute respiratory distress syndrome. Two additional variables, clinically documented pneumonia and the logistic organ dysfunction, were selected by the Fine and Gray model.

**Conclusion:**

The Fine and Gray model appears of interest when predicting mortality in ICU patients. It is closely related to the logistic model, through direct modeling of times to death, and can be easily extended to model non-fatal outcomes.

## Introduction

Mortality in intensive care unit (ICU) patients remains high. The estimated mean in France is about 15% for ICU mortality and 6–25% for hospital mortality after ICU discharge [1], yielding a hospital mortality rate of 20–30%, with substantial variations across studies. Reported factors associated with ICU mortality are partly conflicting. Differences in the statistical methods used to estimate mortality and to identify prognostic factors may contribute to these discrepancies. For instance, the outcome of interest could be hospital mortality, ICU mortality, or mortality at a specific time point (e.g. 14 days, 30 days, 60 days, or three months after ICU admission). Furthermore, some studies determine the prevalence of death and others determine the incidence of death. Prevalence of death is estimated from crude mortality ratios (number of deaths divided by number of ICU admissions), and then logistic analysis is used to identify prognostic factors. Incidence studies estimate survival using the Kaplan–Meier method and then look for prognostic factors in Cox models. Many studies use both approaches, determining survival time distributions by the Kaplan–Meier method and then looking for prognostic factors using logistic models [2].

The main argument against using survival methods to analyze ICU mortality or hospital mortality pertains to censoring. Indeed, the Kaplan–Meier method and Cox model assume that censoring is non-informative (for instance, that the survival time of an individual patient is independent of censoring). In other words, patients discharged alive from the hospital must be representative of all other individuals who have survived to this time of discharge but who are still in hospital. In this case, the distribution of the censoring time is unrelated to the distribution of the survival time, so that censoring is 'non-informative' about the mortality pattern of the population. This is likely to be true if the censoring process operates randomly, which is usually the case when mortality is assessed at a point in calendar time (for instance, on 1 January 2005), provided that this time point is selected before the study is initiated.

**Figure 1:**
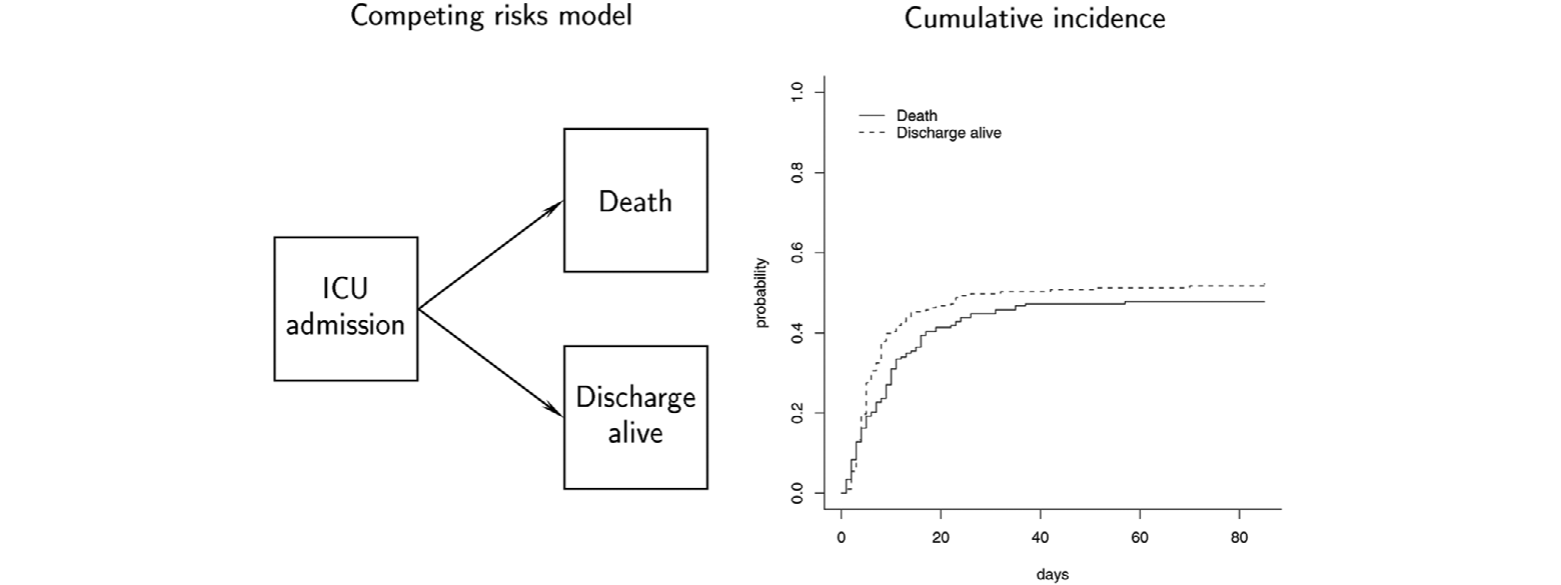
Modelisation of ICU data in the setting of competing risks. Competing risks model (left). Cumulative incidence function of hospital death and being discharged alive (right). ICU, intensive care unit.

However this assumption cannot be made if, for example, the survival time of an individual is censored, being withdrawn as a result of a deterioration or an amelioration in his/her physical condition. This is probably the case in the ICU where patients are discharged alive, and thus withdrawn from the survival analysis, because they need no more intensive care, usually due to amelioration or deterioration of their vital conditions. Patients are therefore discharged alive (censored) because they have a lower risk or higher risk of hospital death than the average. These patients are therefore not the same patient population as those who stayed within the hospital. Resulting censoring is 'informative', meaning that censoring carries information about or depends on the survival time. In other words, informative censoring defined a competing risk, given that discharge from the hospital affects the probability of experiencing the event of interest (death before discharge) (Figure 1). In this setting, standard survival methods are no longer valid, and specific methods need to be considered.

Logistic regression, which is widely used to model hospital mortality, is well suited to the described setting. Nevertheless, logistic modeling has been reported to cause loss of information, because it ignores the time to death and the length of hospital stay [3,4]. Specific statistical approaches dedicated to competing risk data, which allow handling of both censoring and time to events, have been proposed [5-7]. They have been applied to ICU data for predicting the occurrence of a non-fatal event in the face of competing mortality [8]. In studies of mortality in ICU patients, since being discharged alive is also a competing risk, these approaches could also apply directly.

This paper was designed to illustrate the use of competing risks approaches for evaluating the prognostic factors on hospital mortality. To this end, we used a sample of 203 cancer patients admitted to an ICU for acute respiratory failure [9].

## Patients and methods

Between 1 November 1997 and 31 October 2002, all adult cancer patients (= 18 years old) admitted to the medical ICU of the Saint-Louis Teaching Hospital, Paris, France for acute respiratory failure were included. Patients in the cohort were followed up until hospital discharge or death. This study has been previously published elsewhere [9] and will now be briefly summarized.

Hospital mortality was the primary endpoint. Standardized forms were used at ICU admission to collect the following: history of autologous or allogeneic stem cell transplantation (SCT), clinically documented lung disease, microbiologically documented invasive aspergillosis, unknown cause of acute respiratory failure, neurological impairment, alveolar hemorrhage, absence of congestive heart failure, acute respiratory distress syndrome (ARDS), neutropenia, logistic organ dysfunction (LOD) score [10], and history of corticosteroid therapy.

## Statistical analysis

All statistical analyses were carried out using the SAS 8.2 software package (SAS Inc, Cary, NC, USA) and the R 2.0.1 software package [11].

To describe hospital mortality, we utilized a competing risks model (Figure 1). First, we computed the cumulative incidence function (CIF) of death over time. At time *t*, the CIF defines the probability of dying in the hospital by that time *t *when the population can be discharged alive. Note that, contrarily to a distribution function that tends to 1, the CIF tends to the raw proportion of deaths, so it is also called a 'subdistribution function'. The CIF has been estimated from the data using the *cmprsk *package developed by Gray [12].

To estimate the influence of baseline covariates on hospital mortality, we then used logistic models that estimated the strength of the association between each variable and death based on the odds ratio. Finally, we used the Fine and Gray model [7], which extends the Cox model to competing risks data by considering the subdistribution hazard (for instance, the hazard function associated with the CIF). The strength of the association between each variable and the outcome was assessed using the sub-hazard ratio, which is the ratio of hazards associated with the CIF in the presence of and in the absence of a prognostic factor. In both logistic modeling and Fine and Gray modeling, prognostic factors were evaluated in univariate and multivariate analyses. Models were fitted using the *lrm *and *crr *routines in the R software package [11], respectively. Variables associated with the primary endpoint (hospital death) at the 10% level on the basis of univariate models were introduced in the multivariate models.

## Results

Of the 203 patients included in the study, 97 patients (47.8%) died in the hospital. The estimated CIF of death was 35.5% on day 14 and was 47.8% on day 60; no additional deaths occurred after day 60 (Figure 1). Table 1 presents hospital mortality according to the main characteristics at ICU admission. Based on univariate logistic models, eight variables were associated with hospital mortality: autologous SCT, clinically documented lung disease, absence of congestive heart failure, neurological impairment, neutropenia, LOD, unknown cause of acute respiratory failure, and ARDS (Table 2). In the multivariate logistic model, four of these variables supplied independent prognostic information at the 5% level: autologous SCT, absence of congestive heart failure, neurological impairment, and ARDS (Table 3).

From univariate Fine and Gray models, the same eight variables were associated with the cumulative incidence of hospital death (Table 2). When a multivariate Fine and Gray regression model was used, six of the eight variables were found to supply independent prognostic information at the 5% level: autologous SCT, clinically documented lung disease, absence of congestive heart failure, neurological impairment, LOD, and ARDS (Table 3). Variables associated with the cumulative incidence of being discharged alive from the hospital were the same as those associated with the cumulative incidence of hospital death, except for autologous SCT in univariate analyses (Table 2) and LOD in multivariate analysis (Table 3).

## Discussion

Competing risks methods have been used in ICU studies to study non-fatal endpoints in the face of competing mortality [13,14]. We have shown that even mortality in ICU patients can be analyzed using these methods where discharges alive compete with hospital deaths. We illustrated the use and interest of the two main statistical models available in this setting; namely, the logistic model and the Fine and Gray model.

Standard survival analyses are not satisfactory for describing ICU-patient mortality over time: the assumption that censoring is independent of the event of interest (death) is violated, since patients discharged alive are not representative of all other patients still in the hospital. The misuse of the Kaplan–Meier method in this setting is well known and the CIFs have been reported as the optimal tools to measure the probability of the outcome of interest over time [5,6]. The logistic model is widely used, but it ignores the exposure times. Moreover, since the logistic regression does not allow the inclusion of time-dependent covariates [15], one could not adjust for exposure time in that model.

A new regression model, based on CIF-associated hazards, has been proposed by Fine and Gray [7] for identifying prognostic factors in this competing risks setting. This model was first used in cancer patients, to predict non-fatal events such as relapses or metastasis, with death prior to these events as a competing risk [16-21]. We proposed to illustrate the use of the Fine and Gray model for explaining hospital mortality in ICU patients, using a specific R routine [11]. Of note, since all patients either died or were discharged alive by the end of follow-up, standard routines from the SAS package would have been used after recoding exposure times of patients who were discharged alive at the largest observed time of death [5].

Actually, the Fine and Gray model is closely related to the logistic model. The logistic regression focuses attention on the prevalence of hospital death as a measure of prognosis in ICU patients. The Fine and Gray model is based on the hazard associated with the CIF, and therefore predicts the cumulative incidence of death, which tends over time to the prevalence of death. It models this hazard over time, incorporating the different exposure times in the ICU (or hospital) ignored by the logistic model.

As a result, the logistic model and the Fine and Gray model differ in terms of measures used to evaluate the strength of association between the prognostic factor and the hospital death. The sub-hazard ratio on which the Fine and Gray model relies could appear difficult to interpret since it relies on the ratio of subdistribution hazards, which are not directly interpretable in terms of probabilities. Nevertheless, the odds ratio estimated from the logistic model is often misinterpreted as a relative risk — although it should not be misinterpreted unless the outcome of interest is rare (e.g. <20–30%) [22]. When analyzing the mortality of specific ICU patients, such as the cancer patients of our series, this is clearly untrue. In that sense, the sub-hazard ratio appears to be a better approximation of the relative risk than the odds ratio [23].

Estimates of the separate effects of covariates on each outcome could be provided by both models. In ICU patients, when only two risks are considered and no patients are lost to follow-up, fitting a logistic model using either death or discharge alive will result in mathematically equivalent models. By contrast, the Fine and Gray model considers the hazard of death over time so that distinct effects, although inter-related, could be estimated, reaching distinct *P *values. Indeed, a deleterious effect on one risk is necessarily associated with a protective effect on the other risk: therefore, increased mortality in a patient subset is necessarily associated with a decreased incidence of being discharged alive. This was illustrated in our series. For instance, in patients with autologous SCT, the cumulative incidence of hospital death was significantly increased and that of being discharged alive was decreased, but not significantly (Table 2).

In this paper we have raised the differences between both the logistic model and the Fine and Gray model. Of note, both models present limitations mostly due to the required underlying assumptions (proportional hazards for Fine and Gray; log-linearity and additive effects of covariates for both models).

Finally, we evaluated the competing risks approach for predicting hospital mortality because being discharged alive competes with the outcome of interest. The same method could be used to predict ICU mortality. As mentioned earlier, competing risks analyses based on either the logistic model or the Fine and Gray model may also be valuable for modeling non-fatal outcomes in ICU patients, such as mechanical ventilation or nosocomial infection, with deaths before the outcome of interest and being discharged alive as competing risks [13].

## Conclusion

When modeling the mortality of ICU patients, we showed that discharge alive defines a competing risks outcome for hospital (or ICU) mortality. Therefore, besides the widely used logistic regression analyses, standard methods for analyzing competing risks data can be used. Although closely related, the models mostly differ in the handling of exposure times.

## Key messages

• 	When estimating the mortality of ICU patients, being discharged alive from the ICU or from the hospital should be considered a competing risk.

• 	Specific statistical approaches for analyzing outcomes competing against other events are of prime interest in the ICU setting.

• 	To depict mortality over time, the CIF should be used.

• 	To predict mortality, the Fine and Gray model, which is based on the subdistribution hazard associated with the CIF, is an alternate to the widely used logistic regression model.

## Abbreviations

ARDS = adult respiratory distress syndrome; CIF = cumulative incidence function; ICU = intensive care unit; LOD = logistic organ dysfunction; SCT = stem cell transplantation.

## Competing interests

The authors declare that they have no competing interests.

## Authors' contributions

MRR and SC conceived the statistical study and drafted the manuscript. MRR performed the statistical analyses. EA conceived and helped to design and coordinate the clinical study. All the authors read and approved the final manuscript.

## Figures and Tables

**Table 1:** Hospital mortality according to the main characteristics of the population at admission to the intensive care unit

Characteristic	Hospital mortality
Overall population	97/203 (47.8%)
Autologous stem cell transplantation	19/29 (65.5%)
Clinically documented lung disease	17/27 (63.0%)
Absence of congestive heart failure	3/25 (12.0%)
Neurological impairment	36/52 (61.2%)
Neutropenia	41/71 (57.8%)
Unknown cause of acute respiratory failure	24/42 (57.1%)
Acute respiratory distress syndrome	29/40 (72.5%)

**Table 2:** Univariate prognostic analyses based on logistic regression and Fine and Gray regression

Variable	Logistic model		Fine and Gray model			
	
	Mortality		Mortality		Discharged alive	
	
	Odds ratio (95% CI)	*P *value	SHR (95% CI)	*P *value	SHR (95% CI)	*P *value
Autologous stem cell transplantation	2.34 (1.03–5.32)	0.043	1.73 (1.07–2.80)	0.025	0.55 (0.29–1.07)	0.077
Clinically documented lung disease	2.30 (1.09–4.87)	0.029	1. 91 (1.06–3.45)	0.032	0.63 50.42–0.95)	0.027
Absence of congestive heart failure	0.12 (0.04–0.42)	<0.001	0.16 (0.06–0.49)	0.001	2.98 (1.95–4.55)	<0.001
Neurological impairment	3.32 (1.69–6.50)	<0.001	2.35 (1.56–3.55)	<0.001	0.38 (0.23–0.61)	<0.001
Neutropenia	1.85 (1.03–3.33)	0.038	1.61 (1.08–2.39)	0.020	0.65 (0.43–0.97)	0.037
Logistic organ dysfunction	1.20 (1.09–1.33)	<0.001	1.16 (1.09–1.24)	<0.001	0.87 (0.81–0.93)	<0.001
Unknown diagnosis	2.19 (1.13–4.26)	0.021	1.86 (1.20–2.87)	0.005	0.59 (0.35–0.97)	0.039
Acute respiratory distress syndrome	3.68 (1.72–7.89)	<0.001	2.08 (1.39–3.09)	<0.001	0.33 (0.19–0.59)	<0.001

CI, confidence interval; SHR, sub-hazard ratio.

**Table 3:** The logistic and the Fine and Gray multivariate regression models including eight variables selected on the basis of univariate analyses

Variable	Logistic model		Fine and Gray model			
	
	Mortality		Mortality		Discharged alive	
	
	Odds ratio (95% CI)	*P *value	SHR (95% CI)	*P *value	SHR (95% CI)	*P *value
Autologous stem cell transplantation	3.51 (1.37–9.02)	0.009	1.77 (1.00–3.14)	0.049	0.46 (0.23–0.95)	0.035
Clinically documented lung disease	2.01 (0.79–5.14)	0.143	2.09 (1.05–4.15)	0.036	0.71 (0.47–1.08)	0.110
Absence of congestive heart failure	0.12 (0.03–0.57)	0.008	0.22 (0.07–0.64)	0.006	2.20 (1.42–3.42)	<0.001
Neurological impairment	2.63 (1.19–5.81)	0.017	1.84 (1.16–2.91)	0.009	0.44 (0.27–0.71)	<0.001
Neutropenia	1.15 (0.53–2.51)	0.721	1.22 (0.73–2.91)	0.450	0.81 (0.50–1.31)	0.390
Logistic organ dysfunction	1.11 (0.97–1.27)	0.133	1.10 (1.00–1.20)	0.040	0.92 (0.85–1.01)	0.065
Unknown diagnosis	1.82 (0.85–3.89)	0.122	1.20 (0.72–2.00)	0.480	0.71 (0.40–1.27)	0.250
Acute respiratory distress syndrome	3.26 (1.42–7.49)	0.005	1.85 (1.21–2.85)	0.005	0.33 (0.19–0.58)	<0.001

CI, confidence interval; SHR, sub-hazard ratio.
